# Functional Dysphagia Presenting With Severe Subjective Swallowing Difficulty Despite Minimal Objective Findings: A Case Report

**DOI:** 10.1155/crot/1173915

**Published:** 2026-07-03

**Authors:** Fumiyuki Goto, Koichiro Nishiyama, Kenji Okami, Koichiro Wasano

**Affiliations:** ^1^ Department of Otolaryngology, Tokai University School of Medicine, Isehara, Kanagawa, Japan, u-tokai.ac.jp

**Keywords:** cognitive behavioral therapy, functional dysphagia, otolaryngology, phagophobia, predictive coding, Rome IV, visceral hypersensitivity

## Abstract

**Background:**

Functional dysphagia is defined as a persistent or recurrent sensation of abnormal bolus passage without demonstrable structural, inflammatory, or major neuromuscular abnormality. Despite its clinical relevance, otolaryngologic reports of this condition remain limited.

**Case Presentation:**

We describe a 71‐year‐old man who presented with progressive swallowing difficulty following a self‐limited pharyngeal infection. He reported severe subjective difficulty swallowing solids and restricted his diet to liquids. Flexible nasolaryngoscopy, videoendoscopic evaluation of swallowing (Hyodo score 1), videofluorographic swallowing study (VFSS), upper gastrointestinal endoscopy, and neurological evaluation were all essentially normal. No aspiration was documented. Functional dysphagia was diagnosed based on the marked discrepancy between symptom severity and objective findings. Conservative management with reassurance and gradual reintroduction of oral intake resulted in progressive recovery over 4 months.

**Conclusion:**

Functional dysphagia should be considered when subjective swallowing difficulty is disproportionate to objective findings. Recognition of this discrepancy prevents overtreatment and supports multidisciplinary management targeting sensory‐perceptual recalibration rather than anatomical correction.

## 1. Introduction

Dysphagia is a common and potentially serious complaint in otolaryngology. While structural, inflammatory, and neuromuscular etiologies are routinely investigated, functional dysphagia—characterized by swallowing difficulty in the absence of organic explanation—remains underrecognized in the specialty [1, 2]. The Rome IV consensus criteria categorize this condition among functional esophageal disorders, defining it as a persistent or recurrent sensation of abnormal bolus transit without structural or major motility disorder, lasting at least 3 months [1]. Although large‐scale epidemiological data specific to functional dysphagia are limited, functional etiologies account for a meaningful proportion of patients presenting with unexplained swallowing complaints after negative structural evaluation, and recognition of the condition in otolaryngology settings has grown alongside broader awareness of disorders of gut–brain interaction [1–3]. Differential diagnosis requires systematic exclusion of structural causes (stricture, malignancy, and eosinophilic esophagitis), major motility disorders (achalasia and esophagogastric junction outflow obstruction), and neurological conditions (cerebrovascular disease, motor neuron disease, and myasthenia gravis), each requiring targeted investigation with flexible laryngoscopy, videofluorographic swallowing study (VFSS), upper gastrointestinal endoscopy, high‐resolution manometry, and neurological assessment as clinically indicated [2, 4].

The central clinical challenge is the mismatch between reported severity and objective findings. Patients describe food sticking in the throat or esophagus, choking episodes, and progressive dietary restriction to safer consistencies, yet flexible laryngoscopy, VFSS, high‐resolution manometry, and upper gastrointestinal endoscopy reveal no explanatory pathology [2, 5]. This discordance is not indicative of diagnostic failure; rather, it constitutes the defining feature of the disorder. Proposed mechanisms include peripheral visceral hypersensitivity—in which afferent thresholds are lowered following mucosal irritation or a prior inflammatory event—and central sensitization, whereby amplified neural processing sustains conscious perception of swallowing‐related stimuli after resolution of the precipitating cause [2, 6]. A biopsychosocial framework has been proposed as the most comprehensive model for understanding functional dysphagia, integrating sensory hypersensitivity with fear‐avoidance behavior—in which anticipatory anxiety about swallowing drives progressive dietary restriction that paradoxically prevents the corrective experiences necessary for symptom resolution [7]. Cognitive‐affective factors such as anxiety, hypervigilance toward swallowing sensations, and phagophobia (fear of swallowing) further perpetuate symptoms through maladaptive attentional and behavioral cycles [1, 7, 8]. The predictive coding framework offers an integrative account: prior expectations of danger bias sensory interpretation such that normal bolus transit variability is consciously registered as threatening obstruction, generating persistent symptom experience even in the absence of any mechanical abnormality [9].

Despite the clinical burden this condition imposes—manifesting as dietary restriction, social withdrawal, and repeated healthcare utilization—reports from otolaryngology specifically describing functional dysphagia remain scarce. The present case illustrates the diagnostic approach and conservative management in a 71‐year‐old man whose severe subjective swallowing complaints were comprehensively investigated and found to have no organic basis, with gradual recovery over four months following reassurance and behavioral guidance.

## 2. Case Presentation

A 71‐year‐old Japanese man was referred to the outpatient otolaryngology clinic with a three‐month history of progressive swallowing difficulty. He reported that the onset had coincided with a self‐limited episode of pharyngeal irritation and mild throat pain that resolved spontaneously within 2 weeks. Despite clinical resolution of the infection, his swallowing complaints persisted and progressively worsened. He described a persistent sensation of food catching or sticking in the mid‐throat region, a tendency to cough during meals, and intense fear of choking on solid foods. Over the preceding 2 months, he had restricted his oral intake to liquids and pureed foods, avoiding all solid textures. He reported marked anxiety around mealtimes and had begun avoiding shared meals with his family.

His medical history included mild hypertension managed with a single antihypertensive agent. He had no history of cerebrovascular disease, neurodegenerative disorder, head and neck malignancy, prior pharyngeal or esophageal surgery, or gastroesophageal reflux disease. He was a nonsmoker and consumed minimal alcohol. He denied odynophagia, hematemesis, melena, unexplained weight loss, and respiratory symptoms consistent with aspiration pneumonia. There was no change in voice quality, no nasal regurgitation, and no limb weakness or other neurological complaints.

On examination, the oral cavity and oropharynx were structurally normal. Cranial nerve examination revealed intact tongue movement, symmetric palatal elevation, a preserved gag reflex, and no signs of lower motor neuron disease. Flexible nasolaryngoscopy under topical anesthesia showed normal mucosal surfaces of the nasopharynx, hypopharynx, and larynx, with intact bilateral vocal fold mobility, no structural lesion, and no narrowing of the upper aerodigestive tract. Dynamic laryngeal movement during voluntary swallowing trials appeared coordinated, with adequate laryngeal elevation and complete epiglottic inversion.

**FIGURE 1:**
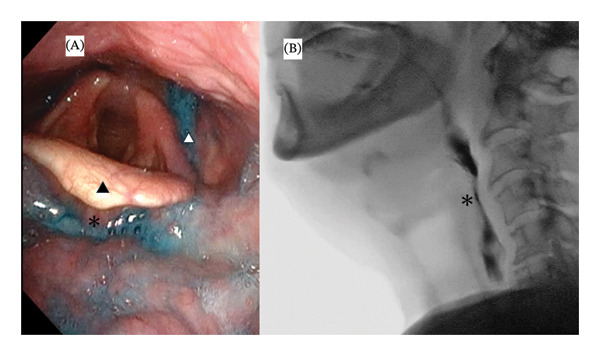
Instrumental evaluation of swallowing demonstrating normal findings despite severe subjective dysphagia. (A) Videoendoscopic evaluation of swallowing using blue‐dyed water. An asterisk (✱) indicates the vallecula, where trace pharyngeal residue was observed (Hyodo score 1), representing a normal physiological variation. A filled triangle (▲) indicates the epiglottis, and an open triangle (△) indicates the left pyriform sinus. No laryngeal penetration or aspiration was observed. (B) Representative static frame from videofluorographic swallowing study (VFSS), lateral view. An asterisk (✱) indicates the esophagus, into which the bolus passed smoothly without obstruction. Bolus transit through the pharynx and into the cervical esophagus was unimpaired, with normal hyolaryngeal excursion and upper esophageal sphincter opening. The trachea is free of contrast, confirming absence of aspiration or laryngeal penetration. Taken together, (A) and (B) demonstrate preserved swallowing biomechanics that are markedly discordant with the patient’s severe subjective complaint, consistent with the diagnosis of functional dysphagia.

Videoendoscopic evaluation of swallowing was performed using a standardized protocol with thin liquid, thickened liquid, and semisolid boluses. Bolus transit appeared efficient without premature spillage or significant residue. Pharyngeal residue was scored using the Hyodo endoscopic swallowing scale [10]: a total score of 1 was recorded, corresponding to trace residue in the valleculae without accumulation—a finding within normal physiological variation. Crucially, no penetration into the laryngeal vestibule and no aspiration were documented at any consistency tested (Figure 1).

VFSS confirmed preserved oral phase transit, adequate hyolaryngeal excursion, coordinated pharyngeal contraction, and normal upper esophageal sphincter opening on both liquid and semisolid bolus challenges. Bolus transit times were within reported normative ranges. No pooling, stasis, or tracheal aspiration was identified. Upper gastrointestinal endoscopy revealed no esophageal stricture, Schatzki ring, mucosal inflammation, or intraluminal lesion. Biopsies of the mid‐esophagus and gastroesophageal junction were histologically normal with no eosinophilic infiltration consistent with eosinophilic esophagitis.

Neurological consultation was obtained to exclude central or peripheral neurogenic etiology. Brain magnetic resonance imaging showed no ischemic lesion, demyelinating plaque, or structural abnormality. Electromyographic assessment of the bulbar musculature was normal. A diagnosis of functional dysphagia was established according to the Rome IV criteria [1]: persistent sensation of abnormal bolus transit for more than 3 months, normal findings on comprehensive objective assessments, and a marked discrepancy between symptom severity and measured swallowing function in the absence of structural, inflammatory, or major neuromuscular disease.

The patient was informed of the diagnostic conclusions in a structured counseling session. Findings from each investigation—including laryngoscopic images, VFSS video recordings, and endoscopic photographs—were reviewed together to demonstrate intact anatomy and unimpaired bolus transit. The nature of functional dysphagia was explained using an accessible description of visceral hypersensitivity: that the swallowing sensory system had become amplified following the prior pharyngeal event, causing normal swallowing stimuli to be consciously experienced as obstructive. The patient was explicitly reassured that no structural disease had been identified and that this absence was protective rather than a diagnostic failure.

No pharmacological treatment or invasive procedure was initiated. The patient was referred to a speech–language pathologist who introduced a structured oral intake program beginning with uniform soft solids and incrementally advancing toward firmer textures over 6 weeks. Premeal diaphragmatic breathing exercises were taught to reduce sympathetic arousal, and attentional redirection strategies—focusing on flavor and social interaction rather than bolus sensation—were practiced under supervision. The patient’s dietary log was reviewed at biweekly intervals. During the first 2 weeks, the patient reported persistent throat awareness with soft solids but denied coughing or any sensation of true obstruction; he described premeal anxiety as still moderately elevated but diminishing compared to the initial presentation. By weeks three to four, he voluntarily extended his dietary range to include minced meat and soft vegetables and spontaneously reported that he was “starting to trust his throat again”—a formulation consistent with progressive recalibration of the fear response rather than passive symptom resolution. By week six, he was tolerating firm solids with only minor residual awareness and had begun attending family meals for the first time since symptom onset. At the 4‐month follow‐up, he reported a near‐complete return to normal oral intake including solid foods, substantially reduced premeal anxiety, and no further episodes of coughing or choking. He had resumed sharing meals with his family. Although no formal psychometric screening tools (such as GAD‐7, PHQ‐9, or HADS) were administered during the workup—reflecting the retrospective, naturalistic nature of this case—the sequential reduction in avoidance behavior, meal‐related anxiety, and subjective distress documented across follow‐up appointments provided clinical evidence of progressive psychological improvement consistent with resolution of fear‐avoidance behavior [7].

## 3. Discussion

### 3.1. Hallmark Feature: Symptom–Finding Discrepancy

**FIGURE 2:**
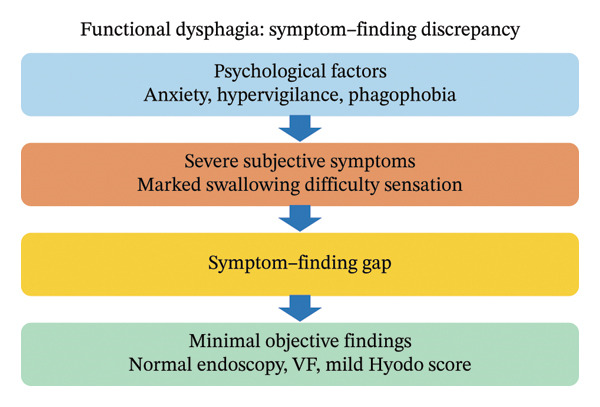
Conceptual model of symptom–finding discrepancy in functional dysphagia. The patient experiences severe subjective swallowing difficulty despite preserved swallowing biomechanics demonstrated by endoscopy and videofluorography. Psychological and cognitive‐affective factors, including anxiety, hypervigilance, and phagophobia, are proposed to modulate symptom perception and amplify interoceptive signals. This model illustrates how altered sensory processing may generate persistent symptoms in the absence of structural pathology.

The defining characteristic of this case is the profound discrepancy between subjective symptom severity and objective findings. The patient described swallowing difficulty severe enough to prompt progressive dietary restriction to liquids, yet VFSS demonstrated normal biomechanics, laryngoscopy showed intact pharyngeal architecture, and videoendoscopic evaluation yielded only a Hyodo score of 1, indicating trace residue within normal variation [10]. This mirrors the Rome IV description of functional esophageal disorders, in which altered sensory perception replaces structural impairment as the dominant pathophysiological driver [1] (Figure 2).

In otolaryngology, such discordance can be diagnostically disorienting. The workup was thorough, but once comprehensive exclusionary assessments returned consistently normal results, further invasive investigation would not have improved outcomes. As demonstrated in noncardiac chest pain, repeated invasive testing after negative workup deepens health anxiety and perpetuates maladaptive illness beliefs without alleviating symptoms [5, 6].

### 3.2. Pathophysiological Mechanisms

The onset coinciding with a self‐limited pharyngeal inflammatory episode, followed by persistent heightened swallowing awareness despite resolution of the precipitating event, is consistent with the visceral hypersensitivity model [2]. Peripheral sensitization lowers activation thresholds of primary afferent neurons via upregulation of acid‐sensitive channels such as TRPV1 and ASIC3 and increased substance P expression within the mucosal afferent network [2, 8]. This creates a state of heightened responsiveness in which routine bolus contact events and minor pharyngeal distension that would otherwise remain subliminal are registered as threatening. Central sensitization then maintains symptoms beyond resolution of the peripheral trigger through increased spontaneous firing rates, expanded receptive fields, and distorted stimulus–response curves within higher‐order sensory processing circuits [6, 8]. Importantly, the patient’s spontaneous reports of intermittent throat awareness between meals—independent of eating—are consistent with this centrally maintained hyperalert state rather than a purely food‐triggered mechanical phenomenon.

The predictive coding framework explains why symptoms persisted robustly despite comprehensively normal findings [9]. Within this model, conscious perception is constructed from a dynamic interplay between top‐down predictions and incoming afferent data. Prior experience of genuine pharyngeal discomfort generated danger priors that biased interpretation of entirely normal mechanosensory input, generating persistent perceptual error signals experienced as obstruction. Crucially, each subsequent swallow provided an opportunity to reinforce rather than extinguish the maladaptive prior because the system was already primed to seek confirmatory evidence of danger and to discount disconfirmatory normal sensory input [9]. This self‐reinforcing architecture explains why symptom intensity escalated over weeks rather than subsiding spontaneously and why reassurance delivered without engaging the underlying perceptual model proved insufficient.

Hypervigilance—sustained deliberate monitoring of each swallow—increased the signal‐to‐noise ratio for pharyngeal sensory input, making benign sensations cognitively salient and distressing [8]. Phagophobia emerged as a behavioral consequence: progressive dietary restriction provided short‐term relief from anticipated choking while simultaneously depriving the patient of the repeated safe swallowing experiences necessary to recalibrate maladaptive expectations [1, 9]. The progressive social withdrawal from shared mealtimes further narrowed the patient’s cognitive attentional set, reinforcing a body‐focused vigilance that maintained central sensory gain.

### 3.3. Differential Diagnosis and Diagnostic Reasoning

Systematic exclusion of organic causes is both necessary and sufficient for establishing the diagnosis [1, 2]. Flexible nasolaryngoscopy excluded proximal lesions including cricopharyngeal hypertrophy and hypopharyngeal malignancy. VFSS excluded oropharyngeal phase deficits and aspiration across bolus types, including solid bolus challenges that can unmask achalasia variants and subtle spasm not apparent on liquid swallows alone [4]. Endoscopy with biopsy excluded eosinophilic esophagitis and malignancy. Neurological assessment excluded cerebrovascular and neurodegenerative causes. The patient’s age necessitated particular vigilance for occult malignancy and early neurodegenerative disease; their consistent absence across multiple modalities provided high confidence in the functional diagnosis [2].

The fear‐avoidance model provides an important organizing framework for understanding why functional dysphagia persists and how it should be treated [7]. Within this model, an initial aversive swallowing experience—in this case, pharyngeal irritation—generates catastrophic appraisal of swallowing‐related sensations as dangerous. This triggers hypervigilance and anticipatory anxiety, which in turn motivate dietary restriction and avoidance behaviors that, while providing short‐term relief, prevent the disconfirmatory experiences necessary to recalibrate maladaptive threat appraisals [7]. Miles et al. proposed a biopsychosocial framework for functional dysphagia assessment and treatment that explicitly integrates fear‐avoidance behavior with sensory hypersensitivity and psychological factors and demonstrated that targeted therapy addressing these interconnected mechanisms can achieve relatively rapid return to normal eating in appropriately identified patients [7]. The present case is consistent with this framework: the patient’s progressive dietary restriction, avoidance of shared meals, and the sequential pattern of symptom resolution through graded exposure all reflect a fear‐avoidance cycle. Therapeutic engagement through graded exposure—providing repeated disconfirmatory swallowing experiences across escalating texture challenges—was the principal mechanism by which the cycle was interrupted.

### 3.4. Management: Rationale and Application

Education and reassurance constituted the first and most fundamental intervention. Reviewing actual instrumental records together with the patient—laryngoscopic images confirming intact anatomy, VFSS video demonstrating unimpaired bolus transit, and endoscopic photographs showing healthy mucosa—transformed abstract assurance into concrete visual evidence that directly challenged the maladaptive priors driving symptom generation [9]. Showing the patient his own normal swallow sequences on video was particularly powerful: it provided a first‐person disconfirmatory experience rather than a clinician’s verbal assertion, which the hypervigilant attentional framework would otherwise readily discount. Reassurance was framed not as dismissal of symptoms but as explanation of mechanism: the swallowing sensory system had been amplified by the preceding pharyngeal event, symptoms were real manifestations of a hypersensitive viscerosensory system, and recovery was achievable through behavioral rehabilitation rather than surgical correction [1, 8].

The speech–language pathology program operationalized graded exposure principles. Beginning with uniform soft textures perceived as low‐threat and systematically advancing toward firmer, more complex foods over 6 weeks, the program generated repeated safe swallowing experiences that progressively recalibrated the patient’s expectation framework [2]. Each successful swallow served as disconfirmatory evidence against the prevailing danger prior, gradually updating the predictive model toward accurate alignment with benign physiological reality. Premeal diaphragmatic breathing exercises counteracted sympathetic arousal—a recognized amplifier of visceral sensitivity—while attentional redirection strategies interrupted hypervigilant monitoring during meals. These techniques align directly with cognitive behavioral principles shown to reduce catastrophic beliefs and maladaptive attentional bias in functional gastrointestinal conditions [1, 8, 9].

The decision to forgo pharmacological neuromodulation reflected progressive clinical improvement under behavioral management alone. In cases where phagophobic avoidance is severe or engagement with behavioral exercises is impaired by unmodulated afferent signals, low‐dose agents such as tricyclic antidepressants or gabapentinoids may reduce afferent gain sufficiently to enable therapeutic engagement [2, 8]. These agents are best presented to patients as visceral desensitizers rather than primary antidepressants, to minimize stigma and maximize adherence. Invasive procedures were appropriately withheld throughout: no structural target had been identified, and pursuing dilation or myotomy without organic pathology would have reinforced the patient’s belief that something mechanical was abnormal—validating rather than challenging the maladaptive predictive model and potentially worsening hypersensitivity through postprocedural sensory changes [2, 9].

### 3.5. Prognosis and Follow‐Up

The 4‐month recovery trajectory—progressive expansion of dietary range, reduction in premeal anxiety, and resumption of social eating—is consistent with the generally favorable prognosis of functional dysphagia following accurate diagnosis and targeted management [1, 2]. The condition does not progress to structural disease or malignant transformation, and this should be communicated explicitly to prevent reescalation of investigation during transient exacerbations driven by stress, sleep deprivation, or intercurrent illness [8].

Follow‐up should prioritize clinical review of dietary function and monitoring for genuine alarm features—rapid weight loss, progressive solid‐to‐liquid dysphagia, hematemesis, or new neurological signs—that would warrant reinvestigation [2]. In the absence of such features, repeat instrumentation adds no safety benefit and risks sustaining healthcare utilization driven by fear rather than clinical necessity [1, 6].

## 4. Conclusion

This case illustrates the presentation, diagnostic approach, and conservative management of functional dysphagia in an elderly patient in an otolaryngology setting. The hallmark feature—severe subjective swallowing difficulty disproportionate to minimal objective findings—requires systematic exclusion of structural and neurological causes followed by a diagnostic pivot toward sensory‐perceptual mechanisms. Visceral hypersensitivity arising from a self‐limited pharyngeal irritation, maladaptive predictive coding sustained by danger priors, and fear‐avoidance behavior collectively maintained symptoms in the absence of structural disease [7]. Effective management under a biopsychosocial framework—integrating education, graded exposure, and behavioral strategies—enabled full recovery without pharmacological or invasive intervention.

Effective management rests on patient education grounded in concrete instrumental evidence, structured behavioral rehabilitation through graded oral intake reintroduction guided by speech–language pathology, and clear communication of the benign prognosis. Avoidance of unnecessary invasive procedures is both therapeutically and ethically central: it protects against iatrogenic harm, prevents reinforcement of maladaptive illness beliefs, and redirects clinical resources toward interventions that address the true sensory‐perceptual basis of the disorder. Increased recognition of functional dysphagia within otolaryngology practice will facilitate earlier accurate diagnosis, appropriate multidisciplinary referral, and prevention of the overtreatment cycles that arise when severe subjective complaints drive repeated investigation despite consistently normal objective findings.

## Funding

This work was supported by JSPS KAKENHI (Grant Number 22K09752).

## Ethics Statement

This study adhered to the Declaration of Helsinki and was approved by the Tokai University Hospital IRB (approval number: 24R086‐001). Written informed consent was obtained from the patient for publication of this case report and accompanying images.

## Conflicts of Interest

The authors declare no conflicts of interest.

## Data Availability

The data that support the findings of this study are available on request from the corresponding author. The data are not publicly available due to privacy or ethical restrictions.
